# Supplementary material for: Experimental investigation of ant traffic under crowded conditions
Source: eLife. 2019 Oct 22;8:e48945. doi: 10.7554/eLife.48945 (PMC6805160; doi:10.7554/eLife.48945)
Supplement: Supplementary file 1. — (Table S1) Parameter estimations for the four functions with weight: To compensate for the discrepancy in the distribution of data points, we adjust the non-linear fitting by giving more weights for points under-represented (i.e.. increase weight ωi for large ki). More precisely, we compute the number Ni of data points for a given density value ki: Ni = #{(kn,qn) with kn = ki}. The weight is then given by ωii1/Ni. Using these weights corresponds to performing a fitting {ki, qi} with qi the average flow at k = ki. Parameter estimation with uniform weight is performed in Table S2. AW: Akaike weights, RSE: relative standard error. (Table S2) Parameter estimations for the four functions without weight. Unlike the Table S1, the parameter estimations use a non-linear regression using all points with the same weight ω = 1. [file elife-48945-supp1.docx]

| Function | Function | *vf* ± CI95 | *k_j_* ± CI95 | α ± CI95 | R^2^ | AW | RSE |
| --- | --- | --- | --- | --- | --- | --- | --- |
| Greenshields | $q=k\cdot vf\cdot\left( 1-\frac{k}{k_{j}} \right)$ | 1.71 ± 0.02 | 25.30 ± 0.28 | NA | 0.79 | 0 | 0.32 |
| Underwood | $q=k\cdot v{fe}^{\frac{-k}{k_{j}}}$ | 2.05 ± 0.04 | 13.89 ± 0.30 | NA | 0.73 | 0 | 0.33 |
| Pipes-Munjal | $q=k\cdot vf\cdot\left( 1-\left( \frac{k}{k_{j}} \right)^{\alpha} \right)$ | 1.70 ± 0.08 | 25.20 ± 0.70 | 1.02±0.11 | 0.79 | 0 | 0.32 |
| Two-phase flow | $q\left( k \right)=\{k\cdot vifk\leq k_{j},k_{j}\cdot vifk>k_{j}$ | 1.34 ± 0.02 | 7.64 ± 0.11 | NA | 0.84 | 1 | 0.30 |

**Table 1**

| Functions | Function | *v_f_* ± CI95 | *k_j_* ± CI95 | α ± CI95 | R^2^ | AW | RSE |
| --- | --- | --- | --- | --- | --- | --- | --- |
| Greenshields | $q=k\cdot v_{f}\cdot\left( 1-\frac{k}{k_{j}} \right)$ | 1.29 ± 0.01 | 194.6 ± 5.43 | NA | 0.83 | 0 | 1.14 |
| Underwood | $q=k\cdot v_{f}e^{\frac{-k}{k_{j}}}$ | 1.28 ± 0.01 | 217.5 ± 7.23 | NA | 0.83 | 0 | 1.14 |
| Pipes-Munjal | $q=k\cdot v_{f}\cdot\left( 1-\left( \frac{k}{k_{j}} \right)^{\alpha} \right)$ | 1.29 ± 0.00 | 16.85 ± 0.04 | 4.16±0.03 | 0.84 | 0 | 1.11 |
| Two-phase flow | $q\left( k \right)=\{k\cdot v_{f}ifk\leq k_{j},k_{j}\cdot v_{f}ifk>k_{j}$ | 1.29 ± 0.00 | 8.14 ± 0.01 | NA | 0.84 | 1 | 1.11 |

**Table 2**
